# Lactose-Containing Dry-Powder Inhalers for Patients with Cow’s Milk Protein Allergy—The Conundrum; A National Survey of Pediatric Pulmonologists and Allergologists

**DOI:** 10.3390/jcm11247346

**Published:** 2022-12-10

**Authors:** Ophir Bar-On, Hagit Levine, Patrick Stafler, Einat Shmueli, Eyal Jacobi, Ori Goldberg, Guy Steuer, Dario Prais, Meir Mei-Zahav

**Affiliations:** 1 Pulmonology Institute, Schneider Children’s Medical Center of Israel, Petach Tikva 4920235, Israel; 2 Sackler Faculty of Medicine, Tel-Aviv University, Tel-Aviv 6997801, Israel

**Keywords:** dry-powder inhalers, lactose, cow’s milk protein allergy

## Abstract

**Introduction:** Several dry-powder inhalers (DPIs) contain lactose which may be contaminated with milk proteins. Confusion exists pertaining to DPI use in patients with cow’s milk protein allergy (CMPA). **Methods:** A computerized survey sent via e-mail to pediatric pulmonologists and allergologists. **Results:** A total of 77 out of 232 (33.2%) doctors replied, of whom 80.5% were pediatric pulmonologists. A total of 69 of 77 (89.6%) were specialists, 37.6% with more than 15 years of experience. The most commonly used DPIs were formoterol + budesonide and vilanterol + fluticasone. A total of 62 out of 77 (80.5%) responders knew these DPIs contained lactose. A total of 35 out of 77 (45.5%) doctors who replied did not know that DPI leaflets list CMPA as a contra-indication to DPI administration. Of these, 4 (11.4%) stated that they would instruct patients with CMPA to stop DPIs, and 7 (20%) would avoid recommending DPIs. A total of 42 out of 77 (54.5%) responders were aware of this warning, yet 13 of these 42 (30.9%) continued to recommend lactose-containing DPIs without hesitation and 18 of these 42 (42.8%) responders prescribed DPIs but considered allergy severity. **Conclusions:** Almost half of certified, experienced pediatric pulmonologists and allergologists were unaware of the warning to administer DPIs to patients with CMPA. Most doctors who do know of this warning still continue to prescribe these DPIs.

## 1. Introduction

Asthma affects millions of people worldwide, both children and adults [1]. Many of these patients additionally suffer from aeroallergen sensitivities and food allergies, including cow’s milk protein allergy (CMPA) [2].

Asthmatic patients use many different types of inhalers. For example, dry-powder inhalers (DPIs) containing bronchodilators and inhaled corticosteroids (ICSs) are frequently used as controllers and reliever therapies in asthma [3].

Many DPIs contain the excipient lactose, an inactive ingredient frequently found in various medications. Lactose is a carbohydrate, milk sugar, and should not be confused with milk proteins. Nevertheless, many web-based medication databases [4] and some DPI leaflets [5,6,7] list CMPA as a contraindication, probably because, during manufacturing, the purification process of lactose could theoretically result in contamination with milk proteins and thus may hypothetically induce an allergic reaction in patients with CMPA.

Approximately 2–3% of infants and young children suffer from CMPA [8]. Approximately 80% of them outgrow it by age five, yet some continue to suffer from CMPA into adult life [9], with a reported prevalence of approximately 0.5%. Some reports stated that CMPA is more prevalent in asthmatic children [10].

Adverse reactions to lactose-containing DPIs in patients with CMPA are seemingly rare, and so some authors have stated that patients with CMPA “need not avoid” DPIs containing lactose [11]. However, a meagre few case reports [12,13,14,15,16] over the last two decades described patients with CMPA who developed an allergic reaction due to lactose-containing DPIs and thus advised against their use in asthmatic subjects with CMPA.

Due to this wide variability, we decided to evaluate the knowledge, experience, and approach of pediatric pulmonologists and allergologists to treating patients with CMPA with lactose-containing DPIs.

## 2. Methods

A computerized Google Forms questionnaire link was sent via e-mail to all participants. The message included a short explanation about the survey and a request to comply. The mail was sent to all pediatric pulmonologists in the country via their society mailing list, which includes 82 doctors. A similar e-mail was sent to all allergologists in the country via their society mailing list, which included 150 doctors (part of which are immunologists). In Israel, allergologists occasionally treat patients with asthma, both adult and pediatric. The e-mails were sent to specialists and fellows indiscriminately. 

The questionnaire was written in Hebrew by the principal investigator, with the aid of clinical pharmacists and the co-authors. Almost all questions were multiple choice questions, some permitting only one answer, while other questions accepted multiple answers, as appropriate. The final two questions were open for free text answers.

The first section of the questionnaire included three basic questions regarding the speciality type, years of experience, and the number of asthma patients seen per week (to assess the overall patient number). The doctors were also asked whether they usually inquire about aeroallergen and food allergen sensitization.

Next, the doctors were asked which dry-powder inhalers they routinely recommended. The following question evaluated doctors’ awareness of the excipients found in dry-powder inhalers. The subsequent section started with a question about the familiarity of doctors with the warning of administering DPIs to patients with CMPA. This question was a split point, i.e., if the reply was “yes”, the participant was forwarded to a specific question, while if the reply was “no”, the participant was forwarded to a different question. Both paths continued to ask about the doctors’ strategy to use or avoid DPIs while treating asthmatic patients with CMPA. The last two questions were “free text”; the first question appeared to participants who chose that they knew about allergic reactions to DPIs, and requested them to describe the event. The final question was an open request for comments.

All the participants answered all the questions. The participant details were anonymized. The results are presented as absolute numbers and percentages. The data are also presented as bar graphs where appropriate. Only descriptive statistics were used.

## 3. Results

The poll was sent via e-mail to 232 doctors, 82 pediatric pulmonologists, and 150 allergologists, of whom only 77 responded. That is, the overall response rate was 33.2%. Most responders, 62 of 77 (80.5%), were pediatric pulmonologists.

Most responders (n = 69, 89.6%) were specialists, i.e., licensed and board certified. Only eight responders (10.4%) were residents in their fields, either pediatric pulmonology or allergy.

Of all the doctors who answered, 29 (37.6%) were specialists with over 15 years of experience, 24 (31.1%) were specialists with 5–15 years of experience, and 16 (20.7%) were young specialists with up to 5 years of experience. Eight residents (10.4%), who were in their pulmonology or allergology subspeciality training at the time of the survey, also responded.

Overall, forty-two doctors (54.5%) reported seeing approximately 1–20 patients per week with a diagnosis of asthma or an asthma-like illness (33 of 62 pediatric pulmonologists, 53.2%, and 9 of 15 allergologists, 60%). Overall, 29 doctors (37.6%) reported seeing approximately 20 to 50 patients per week with a diagnosis of asthma or an asthma-like illness (25 of 62 pediatric pulmonologists, 40.3%, and 4 of 15 allergologists, 26.7%). Overall, three doctors (3.9%) reported seeing more than 50 patients with asthma or an asthma-like illness per week (2 of 62 pediatric pulmonologists, 3.2%, and 1 of 15 allergologists, 6.7%). Three additional doctors (3.9%) reported they do not see patients with a diagnosis of asthma at all (2 of 62 pediatric pulmonologists, 3.2%, and 1 of 15 allergologists, 6.7%). Two of these three doctors were residents, which probably meant they did not see patients by themselves; the last doctor of the three was an experienced pediatric pulmonologist, and thus we regarded his response as a selection/technical error.

We asked all participants about their routine medical patient interviews in regards to allergies, specifically aeroallergens, and food allergens, including cow’s milk protein allergy (CMPA) specifically, and lactose intolerance. A total of 11 (14.2%) out of all the doctors reported they inquired about aeroallergen sensitization. In total, 53 (68.8%) out of all the doctors reported they inquired about aeroallergen sensitization and food allergies, including CMPA specifically. Nine (11.7%) doctors stated that they asked about aeroallergen sensitizations, food allergies, including CMPA, and lactose intolerance. Two (2.6%) doctors reported they asked only about lactose intolerance, and only one (1.3%) doctor reported that they asked only about CMPA during a routine interview. Finally, one doctor (1.3%) out of all doctors who responded stated that they did not routinely inquire about allergies during history taking. To summarize, overall, 73 of 77 doctors (94.8%) who replied reported they asked about aeroallergen sensitization and 63 (81.8%) of all doctors who replied also asked about food allergies, including CMPA specifically.

**Figure 1:**
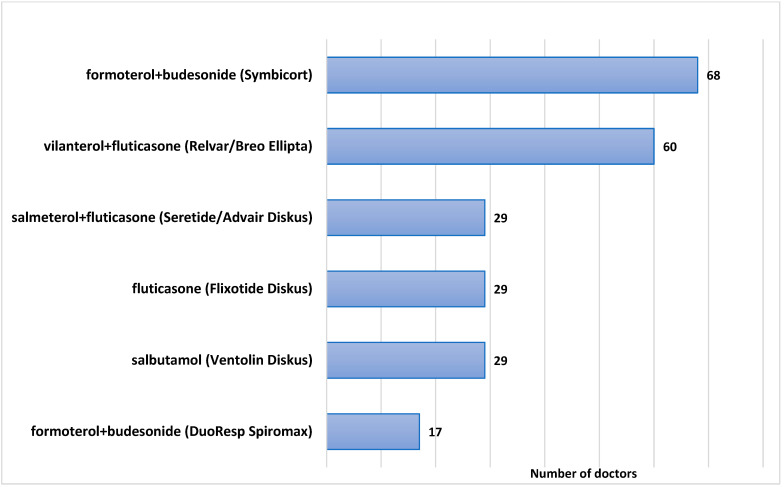
Frequently prescribed Dry-Powder Inhalers for asthma.

When asked about Dry-Powder Inhalers (DPIs) in all dose ranges, that doctors routinely and frequently recommend for patients with a diagnosis of asthma or an asthma-like illness, 68 doctors (88.3%) stated that they routinely recommend a combined long-acting beta-agonist (LABA) plus an inhaled corticosteroid (ICS) inhaler, such as formoterol + budesonide (branded as Symbicort^R^ Turbuhaler^R^); 60 (77.9%) stated that they normally recommend an ultra-LABA/ICS combination of vilanterol + fluticasone furoate (branded as Breo^TM^ or Relvar^R^ Ellipta), and 17 doctors (22%) recommend DuoResp Spiromax, also a combined LABA and ICS inhaler, containing formoterol and budesonide. A total of 29 doctors (37.6%) recommend salbutamol, a short-acting beta-agonist (SABA), in a dry-powder inhaler formulation (branded as Ventolin^R^ Diskus), 29 doctors (37.6%) recommend fluticasone (Flixotide) in a discus dry-powder inhaler formulation, 29 doctors (37.6%) recommend a combined LABA and ICS discus (salmeterol + fluticasone), branded as Seretide^R^ or Advair^R^. The cumulative percentages mentioned are larger than 100% since multiple answers were allowed, and most doctors indicated they recommend more than one type of inhaler on a regular basis. The results are displayed in Figure 1. Several metered dose inhalers (MDIs) also appeared in the responses, such as salbutamol (Ventolin), fluticasone (Flixotide), formoterol + fluticasone (Flutiform), and formoterol + beclomethasone (Foster), but for the purpose of this study, these MDIs were disregarded.

**Figure 2:**
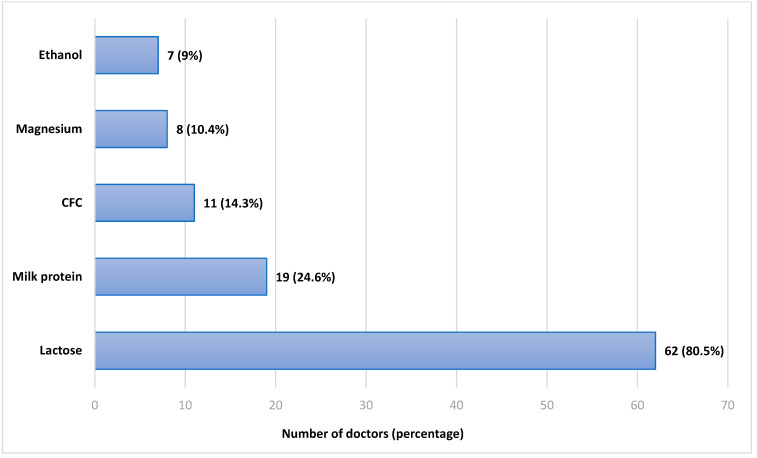
Responses regarding the excipients in Dry-Powder Inhalers. CFC—Chlorofluorocarbons.

The doctors were then asked if they knew what excipients, that is, the non-active ingredients, are routinely found in dry-powder inhalers. Sixty-two doctors (80.5%) indicated they knew that DPIs contain milk sugar (lactose). Nineteen doctors (24.6%) indicated they knew that DPIs contain milk protein. Some responders indicated that DPIs contained certain excipients which they do not, as follows: eleven doctors (14.3%) indicated they knew that DPIs contain CFC (Chloro-Fluoro-Carbons); eight (10.4%) indicated magnesium, and seven (9%) indicated ethanol. The results are displayed in Figure 2. The cumulative percentage is larger than 100% since multiple answers were allowed, and most doctors marked more than one excipient.

The next question was a major one and inquired whether doctors are familiar with the warning written in DPI leaflets and web-based drug databases regarding DPI administration to patients with cow’s milk protein allergy. It was phrased as follows: “in the medication leaflet of some dry-powder inhalers, a warning exists about administration to patients who have cow’s milk protein allergy; did you ever hear about this warning?”. In total, 35 doctors (45.5%) answered that they had never heard about this warning and 42 (54.5%) answered that they knew and heard about this contraindication.

**Figure 3:**
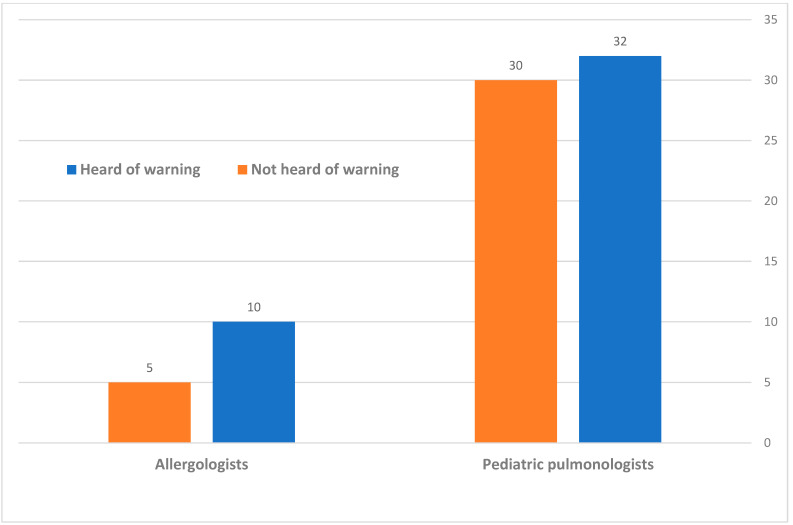
Division of the awareness to the warning of Dry-Powder Inhaler administration in CMPA, according to speciality.

The sub-divisions of these responses, according to the speciality, are displayed in Figure 3. The pediatric pulmonologists (62 total) were fairly equally divided between those who have heard (32 doctors, 51.6%) to those who have not heard (30 doctors, 48.4%) of this warning; A total of 10 of the 15 (66.6%) of the allergologists who responded were aware of this warning.

Of the 42 doctors who confirmed they were familiar with the warning of milk proteins within DPIs, 13 doctors (30.9%) stated that they still continued to recommend and prescribe DPIs to patients known to be allergic to the cow-milk protein, despite this clear contraindication to administering dry-powder inhalers to patients allergic to milk protein. Eighteen doctors (42.8%) stated that they continued to recommend and prescribe DPIs to these patients, but they took the allergy severity into consideration. Eleven doctors (26.2%) stated that they stopped prescribing DPIs to patients with cow’s milk protein allergy once they learned of this warning. 

Of the 35 doctors who replied that they had not heard of the warning, 4 doctors (11.4%) stated that they would now stop prescribing DPIs for patients with cow’s milk protein allergy and think of an alternative treatment. Seven doctors (20%) stated that they would not start DPIs for these patients but rather would find an alternative. Nine (25.7%) doctors reported they would continue prescribing DPIs for patients with cow’s milk protein allergy, despite this warning. Fifteen doctors (42.8%) stated that they would consider stopping DPIs in cow’s milk protein allergic patients, depending on their personal allergy levels.

Finally, we asked all doctors to recall, to the best of their knowledge and memory, if any of their patients who suffer from cow’s milk protein allergy and have received lactose-containing DPIs, developed an allergic reaction. One doctor (1.3%) did not respond to this question. However, the majority of responders, 54 doctors (70.1%), answered that they were not aware of any allergic reactions that occurred to any of their patients who received DPIs. Fifteen doctors (19.5%) answered that they did not prescribe DPIs to these patients. Seven doctors (9%) answered that they did prescribe DPIs in the past, and there were suspicious allergic reactions. Five of these doctors elaborated with free text and described the allergic reactions as a “mild worsening of asthma symptoms”; no comment mentioned a severe systemic allergic reaction that required systemic steroids or epinephrine administration. 

## 4. Discussion

The most striking result of our survey is the fact that approximately half of the certified, experienced pulmonologists and allergologists surveyed, who regularly treat asthmatic patients, declared that they had never heard of the warning to administer lactose-containing DPIs to patients with known CMPA. This contraindication is clearly stated in the medical leaflet of these inhalers and also appears on internet-based medication databases, although occasionally in confusing phrasing. Interestingly, most doctors, including those who declared they were aware of this warning, stated that they continue to recommend lactose-containing DPIs without hesitation to patients with CMPA. On the other hand, some doctors, after participating in this survey, announced they would change their practice and, from now on, would avoid lactose-containing DPIs in patients with CMPA.

None of our survey participants, with a collective experience of hundreds of years, and tens of thousands of patients, reported they were aware of any systemic adverse effects, especially not anaphylactic reactions, in patients with CMPA who received lactose-containing DPIs. Similarly, in the literature, there are just a few reports about such events.

Nowak-Wegrzyn [12] et al. in 2004 wrote a letter to the JACI editor, reporting about an 8-year-old boy with proven severe CMPA who used Advair Diskus, a lactose-containing DPI, without any adverse events for several months. However, upon switching to a new batch, he developed chest tightness which required anti-allergic medication; this was further proven by a supervised challenge that eventually also required intramuscular epinephrine. They obtained the powder from that specific inhaler and powder from other lactose-containing DPIs and tested them for the presence of milk proteins using silver staining and via the immuno-labeling of monoclonal anti-β-lactoglobulin, anti-α-casein, and anti-β-casein antibodies. They indeed demonstrated the presence of milk proteins in all DPIs, with wide lot-to-lot variability. They concluded that the purification process of lactose might have unpredictable protein contamination and therefore advised to avoid, or at least “use with caution” lactose-containing DPIs in patients with CMPA.

Morisset et al. [13] described an adult female with CMPA who presented with asthma exacerbation after exposure to a lactose-containing DPI (formoterol). They concluded that there was a risk of anaphylaxis in patients with CMPA who use lactose-containing DPIs.

Sa et al. [14] reported a 10-year-old boy with known CMPA, who also had asthma symptoms, and thus was started on a fluticasone lactose-containing DPI. Firstly, this administration caused peri-oral urticaria. After a second administration, he developed bronchospasms requiring systemic steroids. They concluded that the contamination of lactose by milk proteins should not be neglected due to the potential risk of allergic reactions.

Robles et al. [15] also reported a case of a 9-year-old child with CMPA who had asthma exacerbation following an administration of a lactose-containing DPI and also concluded that lactose-containing inhaled medications should not be administered to patients with CMPA.

Morikawa et al. [16] described a 6-year-old female with CMPA who developed an anaphylactic reaction after inhalation with Inavir^R^ (laninamivir) to treat the flu. An analysis with a skin-prick test and silver staining of the powder, plus Western blotting, proved the presence of trace amounts of β-lactoglobulin in the lactose excipient. They concluded that lactose-containing DPIs may trigger an allergic reaction in patients with CMPA.

Besides these five reports, despite a meticulous search of PubMed, Google Scholar, and other databases (using keywords such as dry-powder inhalers, lactose, milk allergy, allergic/anaphylactic reaction, and others), we did not find any other case reports describing a possible adverse reaction to milk proteins contaminating lactose in DPIs. Therefore, considering the large number of patients worldwide with CMPA who have most probably received DPIs, this is indeed an extremely uncommon phenomenon, or perhaps an under-reported one.

Spiegel et al. [17] performed a chart review of 8418 asthmatics, of whom 278 had CMPA. Of these, 21 took lactose-containing DPIs and were exposed to a total of 616 inhalers during a total of 715 months. According to the charts, they did not identify any reaction attributable to inadvertent milk protein exposure through these DPIs. Their data suggested that allergic reactions in patients with CMPA taking these DPIs are rare. They concluded that the “watchful vigilance for reactions, not avoidance of these medications, is appropriate”.

In our opinion, this strategy of watchfulness is unacceptable in potential life-threatening allergic reactions. Coinciding with this, current inhaler leaflets clearly state that a severe hypersensitivity to milk proteins is a contraindication to administering lactose-containing DPIs, yet our survey of professional physicians who regularly treat asthmatic patients indicated there was insufficient awareness of this possible hazardous effect. More so, many physicians know of this warning yet choose to ignore it. With all this considered, true life-threatening anaphylactic reactions to these inhalers are apparently very rare, possibly because pharmaceutical companies have improved the lactose purification process over the years or due to exposure route variability, i.e., the inhalation of milk proteins is different from the ingestion of milk proteins and theoretically less immunogenic and allergenic.

Our study has limitations, firstly being a questionnaire study, which relies on the memory of past events. Additionally, only a minority of allergologists answered the study, predisposing the study to a response bias, as the answers might not represent this group. However, overall, the majority of participants in our study were pediatric pulmonologists, with a high rate of response.

To conclude: lactose-containing DPIs may theoretically occasionally contain milk proteins and are therefore officially contraindicated for patients with severe CMPA. In common practice, as evident from our single-nation survey of certified pulmonologists and allergologists, approximately half were unaware of this warning and continued to prescribe these DPIs to patients with CMPA. More so, many doctors who knew of the warning chose to ignore it. Consequently, innumerable patients with CMPA have received these inhalers in recent years. Remarkably, the medical literature of the past 20 years, and the collaborative experiences of our surveyed doctors, contained just a few single reports describing adverse reactions, suggesting that the true contamination of lactose with milk proteins causing anaphylactic reactions is either indeed very rare, under-reported, or confounded by varied allergic phenotypes. Clear, updated regulatory statements should be re-published, and larger studies are required to further assess the safety of DPIs in patients with CMPA.
